# Improved estimates for the role of grey matter volume and GABA in bistable perception

**DOI:** 10.1016/j.cortex.2016.08.006

**Published:** 2016-10

**Authors:** Kristian Sandberg, Jakob Udby Blicher, Simon Hviid Del Pin, Lau Møller Andersen, Geraint Rees, Ryota Kanai

**Affiliations:** a Cognitive Neuroscience Research Unit, CFIN, Aarhus University, Aarhus, Denmark; b Hammel Neurorehabilitation and Research Centre, Aarhus University Hospital, Hammel, Denmark; c Institute of Cognitive Neuroscience, University College London, London, United Kingdom; d Center of Functionally Integrative Neuroscience (CFIN), Aarhus University, Aarhus, Denmark; e Consciousness Lab, Institute of Psychology, Jagiellonian University, Krakow, Poland; f NatMEG, Department of Clinical Neuroscience, Karolinska Institutet, Stockholm, Sweden; g School of Psychology, Sackler Centre for Consciousness Science, University of Sussex, Brighton, United Kingdom; h Department of Neuroinformatics, Araya Brain Imaging, Tokyo, Japan

**Keywords:** Bistable perception, Structure-from-motion, Grey matter volume, Gamma aminobutyric acid (GABA), Magnetic resonance spectroscopy

## Abstract

Across a century or more, ambiguous stimuli have been studied scientifically because they provide a method for studying the internal mechanisms of the brain while ensuring an unchanging external stimulus. In recent years, several studies have reported correlations between perceptual dynamics during bistable perception and particular brain characteristics such as the grey matter volume of areas in the superior parietal lobule (SPL) and the relative GABA concentration in the occipital lobe. Here, we attempt to replicate previous results using similar paradigms to those used in the studies first reporting the correlations. Using the original findings as priors for Bayesian analyses, we found strong support for the correlation between structure-from-motion percept duration and anterior SPL grey matter volume. Correlations between percept duration and other parietal areas as well as occipital GABA, however, were not directly replicated or appeared less strong than previous studies suggested. Inspection of the posterior distributions (current “best guess” based on new data given old data as prior) revealed that several original findings may reflect true relationships although no direct evidence was found in support of them in the current sample. Additionally, we found that multiple regression models based on grey matter volume at 2–3 parietal locations (but not including GABA) were the best predictors of percept duration, explaining approximately 35% of the inter-individual variance. Taken together, our results provide new estimates of correlation strengths, generally increasing confidence in the role of the aSPL while decreasing confidence in some of the other relationships.

## 1 Introduction

Across a century or more, ambiguous stimuli have been studied scientifically because they provide a method for studying the internal mechanisms of the brain while keeping the external stimulus constant. Bistable (or multistable) perception occurs when the brain faces a stimulus that can be interpreted in two (or more) ways. Examples include the Necker cube where a set of lines can be interpreted as two differently oriented cubes (Necker, 1832), Rubin's face/vase where a stimulus can be seen as either a face or a vase depending on which part of the stimulus is perceived as the background (Rubin, 1915), and binocular rivalry where a different image is presented to the same part of the visual field of each eye (Breese, 1899). In recent years, several studies have reported neural correlates of perceptual reversals during bistable perception, and various models have been proposed to explain the phenomena. In particular, an increased focus on two particular brain characteristics, grey matter (GM) volume of specific areas in the parietal lobe and the relative gamma aminobutyric acid (GABA) concentration in the occipital lobe, has become apparent, and there are indeed several reasons to believe that both factors are involved in perceptual reversals.

For example, inhibitory (GABAergic) neurons may have a key role relating to cross-inhibition between representations in multiple models of bistable perception (e.g., Noest et al., 2007, Wilson, 2003). The predictions of such models (e.g., Wilson, 2007) are consistent with a recent finding in an intermittent binocular rivalry magnetoencephalography (MEG) study (Sandberg et al., 2014a, Sandberg et al., 2014b). Here, a face was presented to one eye and a grating was presented to the other for brief durations on each trial, and the participants reported their perception, which typically remained stable across tens of trials before alternating. Across ten trials (of grating perception) before perception reversed to face perception, the amplitude of the face-specific M170 component increased, consistent with the prediction that adaptation of GABAergic neurons responsible for cross-adaptation lead to decreased suppression of the face stimulus. If reversals were simply related to random fluctuations or adaptation neurons representing the perceived stimulus, this modulation of activity related to the unperceived stimulus should not have been observed.

In the same study, parietal activity was also observed immediately before a reversal, suggesting a role of the parietal lobe in initiating such reversals. Previous electroencephalography (EEG), functional magnetic resonance imaging (fMRI) and transcranial magnetic stimulation (TMS) studies have also suggested a role of the (superior) parietal lobe either by observing parietal activity at the time of reversals or by manipulating the alternation rate by stimulating an area within the superior parietal lobule (SPL) (Britz and Pitts, 2011, Carmel et al., 2010, Lumer et al., 1998).

**Fig. 1:**
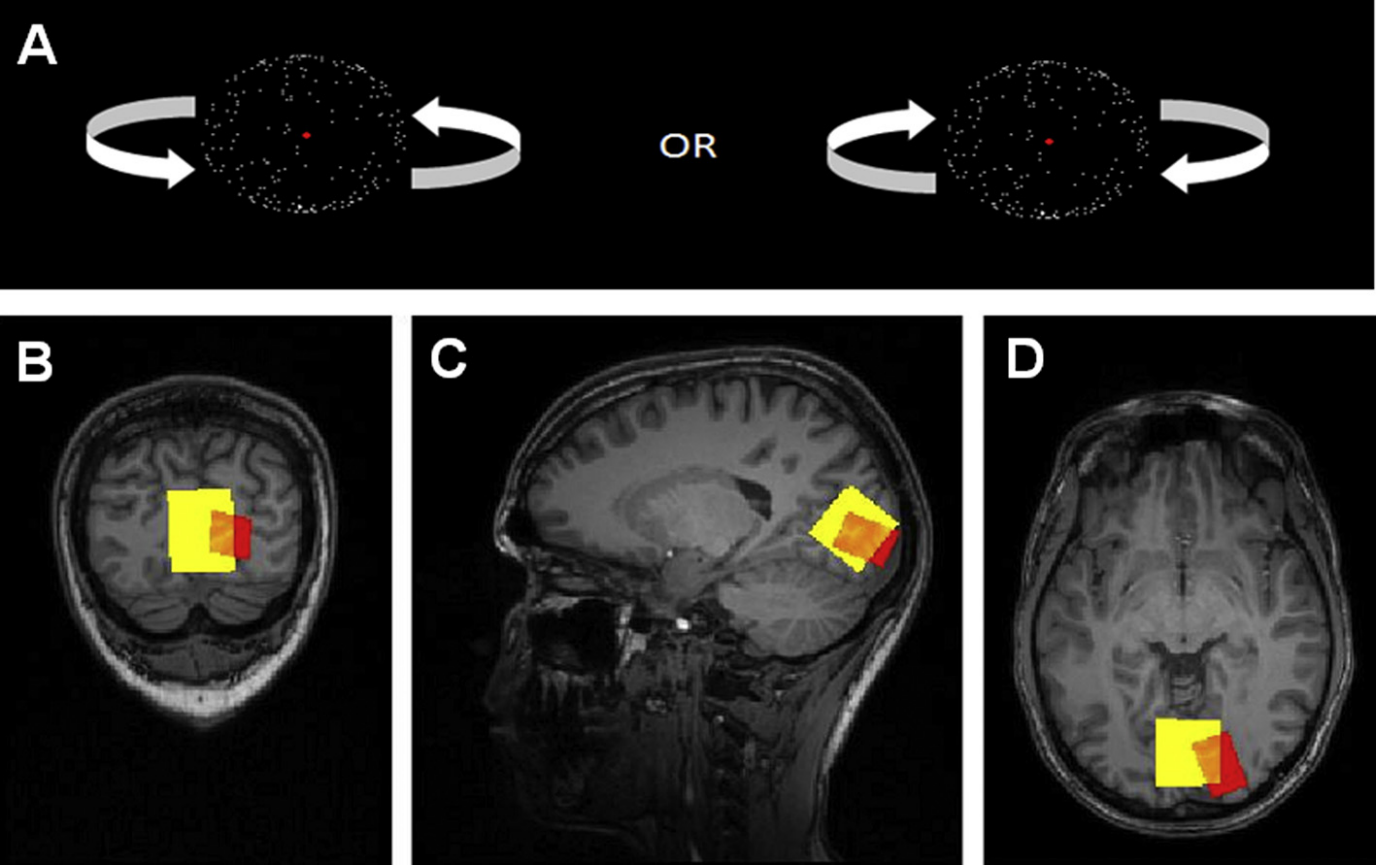
Paradigm. (A) Structure-from-motion (SFM). An ambiguous sphere, which could be perceived as rotating in one of two directions (as indicated), was presented on each 60 sec trial. Participants reported the perceived direction of rotation continuously and the percept duration was examined. (B–D) Example of voxel placement for one participant for the large (yellow) and small (red) voxels used for GABA magnetic resonance spectroscopy (MRS) presented in coronal (B), sagittal (C), and axial (D) planes.

Three recent studies have provided evidence for the roles of parietal GM volume and occipital GABA concentration. Specifically, van Loon et al. (2013) reported a positive correlation between mean percept duration during bistable perception and occipital cortex GABA as measured by magnetic resonance spectroscopy (MRS) whereas Kanai, Bahrami, and Rees (2010) reported negative correlations between the GM volume of left and right posterior SPL and percept duration. In a reanalysis of the same data [informed by another study (Carmel et al., 2010)], Kanai, Carmel, Bahrami, and Rees (2011) also identified a positive correlation between GM volume of an anterior area within the SPL and percept duration. Here, we report a replication analysis of these findings using a paradigm similar to those of the original studies.

van Loon et al. (2013) used three types of stimuli, but found the strongest relationship between effect for structure from motion (SFM) and occipital GABA. In the Kanai et al. studies, SFM was also used. In the study reported here, we use the same stimulus type. In the SFM paradigm, a set of dots are presented in motion in a manner that is compatible with the rotational movement of a single, transparent object (typically a sphere), and participants report perceiving such an object. However, as there is no depth cue, one set of dots can be perceived as either forming the nearest or the farthest surface of the stimulus, and the brain switches between interpretations spontaneously over short intervals of time (from seconds to a few tens of seconds), hence the bistability (Fig. 1A).

It should be noted that the data for the present study were collected before the van Loon et al. (2013) study was reported, and for that reason, there are some differences in experimental details. As such, the present study should not be considered a direct replication attempt of that study, but the studies are nevertheless so similar that the present analysis may be considered more than simply a conceptual replication. All differences are mentioned in the Methods section, and the important differences are discussed in the Discussion section. There are only minor differences between the present study and Kanai et al., 2010, Kanai et al., 2011.

## 2 Methods

The MRS data for the large occipital voxel has been used in two previous articles (Near et al., 2014, Sandberg et al., 2014b). Parts of the methods description below are adapted from the latter publication. The number of participants, 37, was determined by these previous studies, and the number was selected because a power of at least 70% is achieved for moderate (*r* = .4) or stronger correlations.

### 2.1 Participants

Only males participated in the experiment as cortical GABA concentration varies with the menstrual cycle in females (Harada, Kubo, Nose, Nishitani, & Matsuda, 2011). 37 healthy young males gave written informed consent to participate in the experiment. They were between 20 and 40 years of age (mean = 25.4, SD = 4.70) and had normal or corrected-to-normal vision. The experiments were approved by the local ethics committee, De Videnskabsetiske Komitéer for Region Midtjylland, Denmark.

The age of the participants is comparable between this and the studies by Kanai et al. (mean = 26.2, SD = 5.27) and van Loon et al. (mean = 22.7, SD = 1.52). Additionally, only males were included in the van Loon et al. study whereas Kanai et al. included both males and females, but regressed out the effect of gender in the analyses.

### 2.2 Structure from motion (SFM)

All 37 participants completed an SFM task (Fig. 1A). An ambiguous rotating sphere comprising 200 full-contrast white dots and a diameter subtending 3.5° was presented against a black background on a 17-inch LCD monitor using PsychToolbox 2 running under MATLAB (Mathworks). The screen resolution was 1024 × 768 pixels and the refresh rate was 60 Hz. The dots moved sinusoidally with an angular velocity of 120°/sec. A red fixation cross (.5° in height and width) was superimposed on the centre of the sphere to aid steady fixation. On each trial, the dots comprising the ambiguous rotating sphere were presented continuously for 60 sec. Participants were instructed to report the perceived direction of the rotation of the sphere by holding down one of two keys. They were also instructed not to press any key when the percept was unclear. Before the main experiment, participants were given practice trials to ensure they understood the task and instructions. Then, they completed 8 runs of trials (a total of 480 sec) for data acquisition to be used in subsequent correlation analyses.

The experimental paradigm was identical to that used by Kanai et al. (in fact the same program was used) with one minor change: The screen refresh rate was set to 60 Hz instead of 75 Hz. As the rotation speed of the sphere was dependent on the refresh rate, this caused the angular velocity to be 120°/sec rather than 151 in the Kanai et al. study. It also meant that each trial lasted 60 sec instead of 48. In the van Loon et al. study, the sphere consisted of a higher number of dots (1850), which could be either black or white. The size of the sphere was not reported. The rotation speed was 80°/sec. As in our study, a refresh rate of 60 Hz and a resolution of 1024 × 768 pixels were used. Reports of perception were obtained in the same manner as in our study. However, in the van Loon et al. study, participants were instructed to attempt to increase the alternation rate on every other trial. As the results were not reported separately, it is unknown if the correlation between occipital GABA and mean percept duration was found for passive or active viewing only, or for both.

### 2.3 Magnetic resonance imaging (MRI)

Participants were scanned on a Siemens Tim Trio 3T MRI-scanner (Erlangen, Germany). A T1 magnetization-prepared rapid gradient-echo (MPRAGE) structural scan (TR/TE 2420/3.7 msec, 1 mm isotropic resolution, scan time 5 ½ minutes) was performed and used for subsequent GABA voxel placement and for voxel-based morphometry (VBM) analyses.

GABA edited MRS was performed using MEGA-PRESS (Edden and Barker, 2007, Mescher et al., 1998). In brief, the methods acquire two different spectra; one with an editing pulse targeting the C3-GABA peak at 1.9 ppm (edit on) and another with the editing pulse symmetrically on the other side of the water peak (7.5 ppm, edit off). By subtracting the two spectra, the C4-GABA peak at 3 ppm (affected by the editing pulse through J-coupling with the C3-GABA protons) becomes visible and can be quantified. As a coupled macromolecule resonance at 3 ppm is also co-edited, and thus contributes to the measured signal, the term GABA+ is often used. By adding the two spectra, instead of subtracting, the Creatine (Cr) peak at 3.0 ppm can be quantified and a GABA+/Cr ratio can be calculated.

For MEGA-PRESS MRS, the scan parameters were TR/TE: 2500/68 msec. Data were obtained for two occipital voxels, a large voxel including large parts of the occipital lobe (but not extending into parietal and temporal lobes) and a small voxel positioned along the calcarine sulcus of the right hemisphere. The large voxel had the dimensions 30 × 30 × 30 mm, and a total of 96 averages (edit on and edit off) were measured, leading to a scan time of 8 min. The small voxel had the dimensions 18 × 18 × 25 mm, and a total of 240 averages were measured, leading to a scan time of 20 min. For the large voxel (Fig. 1B–D), the calcarine sulcus and the parieto-occipital sulcus were identified bilaterally. The voxel was placed so that it covered the calcarine sulcus bilaterally and so that one edge was aligned with the parieto-occipital sulcus and then shifted as far towards the tentorium cerebelli and the occipital pole as possible. Care was taken to avoid including the scalp and/or the tentorium cerebelli in the voxel. For the small occipital voxel (Fig. 1B–D), the calcarine sulcus was identified and the voxel placed along this.

The voxel used in the van Loon et al. (2013) study had the dimensions 30 × 25 × 20 mm and was centred bilaterally on the calcarine sulcus. The volume of the voxel was 15 cm^3^ whereas the volumes of our large and small voxels were 27 cm^3^ and 8.1 cm^3^. The voxel size used in the van Loon et al. study thus falls in-between the sizes of the voxels used in this study being numerically closer to the small, but proportionally being around half the size of our large voxel and around twice the size of our small voxel. In both studies, the calcarine sulcus was used in voxel placement. However, our large voxel also covered a large proportion of the remaining occipital lobe, whereas the small voxel covered mainly the majority of the calcarine sulcus and extended only into neighbouring areas. It is worth noting that unlike the large voxel and the voxel used by van Loon et al., the small voxel did not include cerebrospinal fluid between hemispheres.

van Loon et al. (2013) reported GABA corrected for GM volume, but did not specify exactly how the correction was performed. As the exact procedure was unknown, we report GABA without GM volume here. However, it should be noted that we also ran all tests using a GM volume correction, and the results were qualitatively unchanged as the corrected and uncorrected values were highly correlated (*r* > .8). The correction was performed by dividing the GABA/CR ratio by the fraction of grey matter within the voxel (GABA/CR/GM).

As GABA levels do not change depending of the time of the day (Evans, McGonigle, & Edden, 2010), the time of the day for the MRS scan was not controlled for between participants. Participants completed the SFM task within one week of the MRS scan of the large voxel. All 36 participants completed this session. The MRS scan of the small voxel was performed 6–9 months later and completed by 22 participants. This session included a re-scan of the large voxel. Using Bayesian analyses, we have previously demonstrated that GABA levels for the large voxel remained stable across this period (Near et al., 2014), and the time between scanning sessions should thus not be considered a confound.

### 2.4 Analysis

#### 2.4.1 Voxel-based morphometry

For VBM, the MR images were first segmented for GM and white matter (WM) by using the segmentation tools in SPM8 (http://www.fil.ion.ucl.ac.uk/spm). Subsequently, we performed diffeomorphic anatomical registration through exponentiated lie algebra (DARTEL) (Ashburner, 2007) for inter-participant registration of the GM images. The registered images were smoothed with a Gaussian kernel (FWHM = 10 mm) and then transformed to Montreal Neurological Institute (MNI) stereotactic space for regression analysis. The only difference between our analysis and the analyses of the Kanai et al. studies was a slightly different kernel for smoothing (FWHM = 10 mm instead of 8 mm), which is unlikely to cause more than very minor differences in results due to the high correlation in GM volume across small distances.

#### 2.4.2 Magnetic resonance spectroscopy

MRS data were analysed by author JUB who was blind to the results of the SFM analysis. Removal of motion corrupted averages, drift correction and phasing of individual MRS data were performed in MATLAB, using the FID-A processing toolkit (https://githib.com/CIC-methods/FID-A) (Simpson, Devenyi, Jezzard, Hennessy, & Near, 2015). Subsequently, the AMARES package (Vanhamme, Sundin, Hecke, & Huffel, 2001) within jMRUI (Naressi, Couturier, Castang, de Beer, & Graveron-Demilly, 2001) software was used to estimate GABA+ from the difference spectra and creatine from the summed spectra. The final results were expressed as the GABA+/Cr ratios. This is comparable to the procedure used by van Loon et al. where GABA was also expressed in relation to creatine.

A visual data quality check was performed in combination with objective quality criteria. For the large voxel, data were excluded if they showed line broadening (line width > 8 Hz) or had high fit uncertainty of the Creatine peak in AMARES (SD/amplitude ratio > .20). For the small voxel a line width up to 10 Hz was accepted due to the slightly lower quality of the data from the smaller voxel (see the paragraph below). 10 of the 72 datasets were excluded (3 large and 7 small voxels) either due to line broadening (1 large and 5 small voxels) or fit uncertainty (2 large and 1 small one small voxel). In the van Loon et al. (2013), data from 4 (of 18) participants were reported as excluded due to low signal-to-noise ratio (SNR) although no specific threshold was reported. Overall, this proportion appears comparable to that of the present study.

SNR was calculated using the difference spectrum following phase adjustment such that the N-acetylaspartate (NAA) peak was upright with a phase of 0°. Signal was calculated as the maximum intensity of the real part of the NAA peak in the phased difference spectrum, and noise was calculated as the standard deviation of the real part of the noise in a signal-free part of the spectrum following a baseline correction to remove any 1st and 2nd order baseline variations. Although a higher SNR was obtained for the large occipital voxel (226) than for the small occipital voxel (117), both SNRs were acceptably high for the detection of GABA.

### 2.5 Statistics

Correlations between log-transformed SFM percept duration, large and small occipital GABA+/Cr ratio, and GM volume of the SPL sites reported in previous publications were tested. GM volume analyses were also performed using a spherical region of interest (ROI) with a 15 mm radius centred on the coordinates reported in previous publications and using a mask ROI consisting of all significant voxels in the Kanai et al. (2010) study.

Data were analysed using the Pearson Product-Moment Correlation, which has five assumptions: 1) That the variables are interval or ratio measurements, 2) that variables are approximately normally distributed, 3) that there is a linear relationship between the two variables, 4) that outliers are kept to a minimum or removed entirely, and 5) that the data are homoscedastic.

All these assumptions were tested. All variables were interval measurements. Due to the nature of the data, normality was expected for all variables, and qq-plot and histogram inspections did not refute this assumption for log-transformed percept duration, GABA+/Cr ratio and GM volume at the exact peaks reported in previous publications. However, the assumption was refuted for two GM volume variables (spherical ROI peak coordinates [−21 −63 61] and [34 −66 34]). These peaks were then replaced by second and third highest peaks within the sphere respectively to avoid using data not appropriate for the analysis. It should be noted that this leads to only marginally different correlation coefficients with no qualitative impact on the conclusions (*r* = −.220 and *r* = −.196 for the correlations meeting the assumptions *vs r* = −.237 and *r* = −.250 for the original correlations). All relationships were linear (see Results). No outliers were observed by visual inspection. From visual inspection, data appeared homoscedastic.

As we were interested not only in evidence against the null, but also for the null, Bayesian analyses are reported in the Results section. We have included an appendix with traditional, frequentist statistics.

#### 2.5.1 Bayesian analyses

The majority of the Bayesian replication tests were performed using R code by Josine Verhagen available at http://www.josineverhagen.com/?page_id=76. In Bayesian statistics, a Bayes Factor (BF) is calculated representing the odds that the observed data occurred under one model versus another, in our case the null hypothesis (H_0_) and an alternative hypothesis (H_1_), where H_0_ postulates that there is no correlation between the measures of interest whereas H_1_ postulates that there is a correlation. If, for instance, the data is 4 times as likely to have occurred under the alternative hypothesis, BF_10_ = 4, also written as BF_01_ = 1/4 (indicating that the data is 4 times less likely to have occurred under the null hypothesis). In this study, we report BF_10_ at all times. Although the BF is continuous, varying conventions exist for the terminology of the strength of evidence. Here, we use the terminology of Wetzels and Wagenmakers (2012) labelling a BF (and 1/BF) between 1 and 3 as anecdotal evidence, between 3 and 10 as substantial evidence, between 10 and 30 as strong evidence, between 30 and 100 very strong evidence, and >100 extreme evidence.

For H_1_, the prior probability distributions (or simply priors) can be informed by the results (the correlation coefficients and number of participants) of the study to be replicated (an informative prior), or it can simply be a uniform distribution from 0 to 1 for expected positive correlations or from −1 to 0 for expected negative correlations. In this study, we generally report BFs using both informative and uniform priors. To perform analyses using informative priors, the data from the study to be replicated was first analysed against a uniform prior. The resulting posterior was then used as the (informed) prior for an analysis based on our data. This means that the resulting posterior from this analysis is identical to the posterior obtained if the data from the original and our study had been analysed together in the first place.

Generally, the BF based on the informative prior should be considered more useful as it reflects more accurately the prior belief that would be reasonable based on the previous studies. However, as we discuss below, the multiple comparison approach used in VBM studies is likely to lead to exaggerated correlation coefficients, and the most realistic prior should thus be shifted slightly toward 0. However, the exact magnitude of this shift is difficult to estimate, and we thus report the BF obtained by using a uniform prior as a more naïve method. It should be noted that a uniform prior without direction (a uniform distribution ranging from −1 to 1) is perhaps the most uninformative prior, and the one-sided priors used here thus carry more information than that.

Spherical ROI replication analyses have been advocated by Kanai (2016) because analyses based on exact coordinates may underestimate effect sizes due to spatial uncertainty of the peak correlation site across samples. Kanai proposed a specific frequentist analysis, and we report the results of this analysis in the Appendix. We also conducted two types of Bayesian analyses using the spherical ROI data. First, we conducted the analysis described above using uniform and informed priors on spherical ROI instead of exact peak data. Whereas the exact peak data is expected to underestimate the correlation, the spherical likely overestimates it slightly due to multiple comparisons, and the true correlation is thus likely somewhere in-between. Second, SPM12 was used to perform posterior probability mapping using the Savage-Dickey-Taylor approximation to the Bayes Factor (Penny & Ridgway, 2013). An Empirical Bayes approach was used to estimate the prior from the spherical ROI data, with the prior mean set to zero and the shape defined as Gaussian, and evidence against the null was examined at the peak correlations within the ROI.

Finally, multiple regression analyses were performed in RStan using the R Rethinking package. A Gaussian prior with a mean of zero and a standard deviation of 10 was used for all parameters. Model evaluation was based on the Watanabe-Akaike information criterion (Watanabe, 2010). WAIC is a Bayesian approach for estimating the out-of-sample expectation, starting with the computed log pointwise posterior predictive density and then adding a correction for effective number of parameters to adjust for overfitting.

#### 2.5.2 Frequentist analyses

For GM volume, *p* < .05 (uncorrected) was set as the criterion for statistical significance for analyses focussing on the exact coordinates reported in previous publications as well as for occipital GABA/Cr + ratio analyses. For spherical ROI and mask ROI analyses, we used *p* < .05 family-wise error rate corrected for the small volume of the ROI as the criterion for statistical significance. All tests for which the direction of the correlation was known were one-tailed.

For any replication analysis (Bayesian or frequentist), the number of participants in the replication sample has a large impact on the probability of success. The number of participants and the *r* of the original studies are presented in Table 1. Power estimates were approximated using the R package pwr. For the three GM volume correlations, power was .998, .993, and .743. For the two GABA correlations, power was .995 and .851. All calculations assumed one-tailed tests. Even if we consider that spatial uncertainty could inflate the power estimates slightly, the power of the study is, over all, more than sufficient.

## 3 Results

Behavioural analyses as well as Bayesian replication analyses are reported in this section. Frequentist analyses are reported in the Appendix (Section 5) following the same overall structure, but without behavioural analyses. In this way, Section 3.2 of the results thus reports Bayesian analyses of the same data used in frequentist analysis in Section 5.1 and so on.

### 3.1 Behavioural analyses

SFM percept duration followed a gamma distribution with a geometric mean of 11.00 sec (95% CI: [9.53; 12.67]). This is somewhat higher than in the Kanai et al. and the van Loon studies where the geometric mean appeared to be in the range of 5–6 sec although no formal test of a difference was conducted as means and variance were not reported in the two other studies (only individual participant data in graphs). When comparing to the Kanai study, the apparent difference in percept duration is a bit surprising as the only difference in the presentation display between the studies was a slight change in rotation speed of the sphere, from 151°/sec to 120°/sec. Another design difference that might explain the different alternation rates is that only males were included in our study as explained in the Methods section. The difference to the van Loon study is not as surprising as participants in that study were instructed to attempt to increase the alternation rate on even-numbered trials, thus decreasing the average percept duration.

### 3.2 VBM analyses

Three types of data selection were used for replication analyses: Using 1) the exact coordinates reported in previous publications, 2) a spherical ROI with a 15 mm radius centred on the coordinates reported in previous publications, and 3) a mask ROI consisting of all statistically significant voxels in the Kanai et al. (2010) study (the threshold for significance was set at *p* < .001, uncorrected). These three types of data selection have advantages/disadvantages, which are discussed in-depth in the Discussion. The most important aspect to note is that the exact coordinate is likely to underestimate the true maximum correlation (as a consequence of small spatial variability across study samples), whereas the ROI peak correlations are likely to overestimate the true maximum (due to random variability within the ROI combined with selective sampling of the peak correlation).

**Fig. 2:**
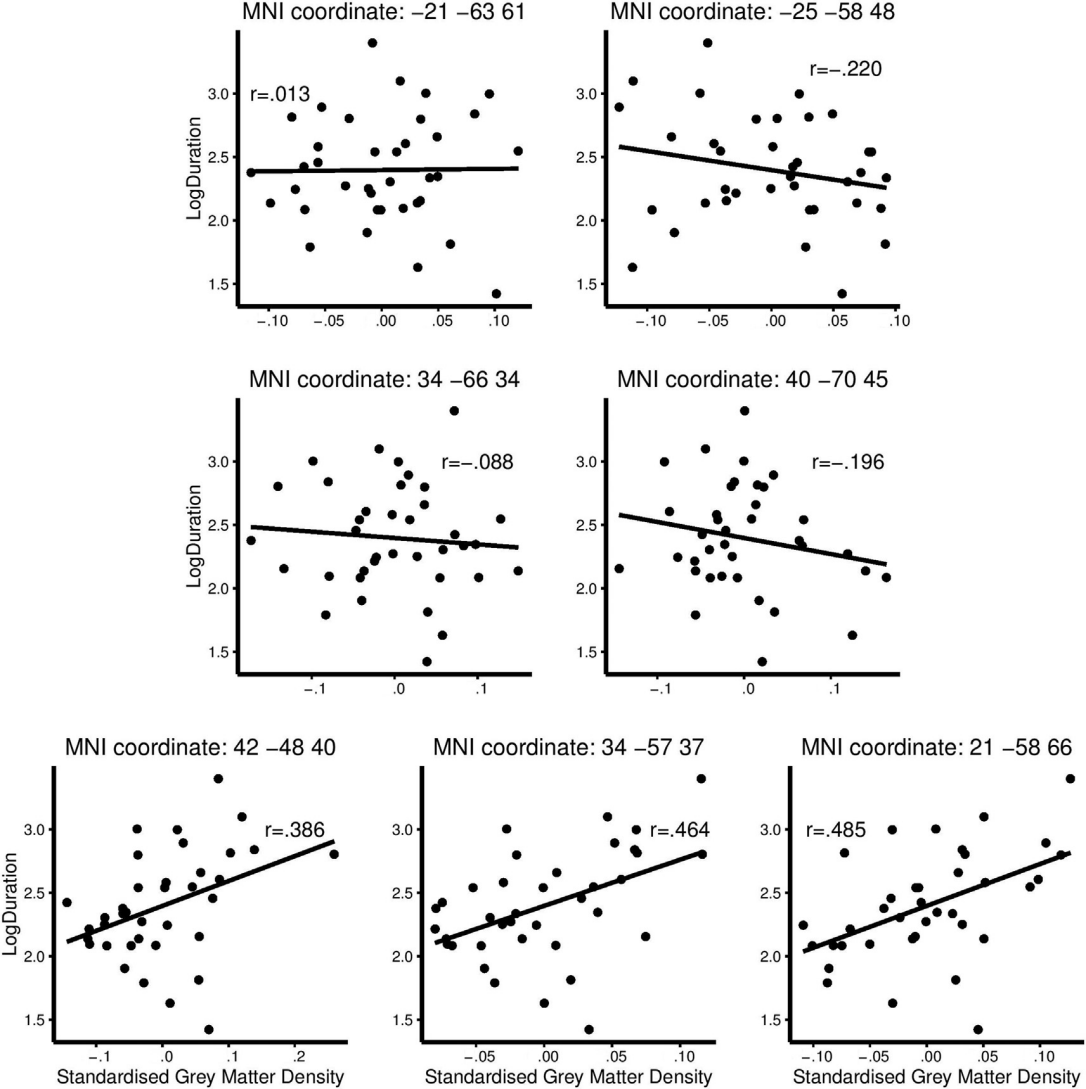
Grey matter (GM) volume correlations. GM volume and mean log-transformed percept duration is plotted for all participants (one point representing one participant) for all coordinates reported in Table 1. Linear regression lines are fitted for all plots.

All correlations are shown in Fig. 2. Overall, no or weak correlations were found for the two posterior coordinates reported in Kanai et al. (2010), yet a moderate correlation was found when using the mask consisting of significant voxels from that study. A moderate correlation was also found at the anterior coordinate reported in Kanai et al. (2011).

For all three data selection methods, analyses were performed using uniform and informed priors. First, a one-sided Bayesian hypothesis test was performed using a uniform prior. As seen in Table 1 (column 8), moderate evidence *for* the null (BF = .2 and BF = .32) was found for the two exact peaks reported in Kanai et al. (2010) whereas the evidence was anecdotal when using the strongest negative correlation within the ROI (BF = .83 and BF = .68). Moderate evidence *against* the null (BF = 5.48) was found for the exact peak coordinate of Kanai et al. (2011), and the evidence was strong when using the peak within the ROI (BF = 20.6). Similarly, strong evidence against the null was found when using the strongest correlation in the Mask ROI condition (BF = 15.4). Taken together, our replication analysis using uniform priors thus generally decreased our belief in a negative correlation between GM volume and SFM percept duration at the peak coordinates reported in Kanai et al. (2010), but at the same time, our belief in a correlation between percept duration and GM volume at a mask ROI coordinate as well as the coordinate reported in Kanai et al. (2011) was increased.

**Fig. 3:**
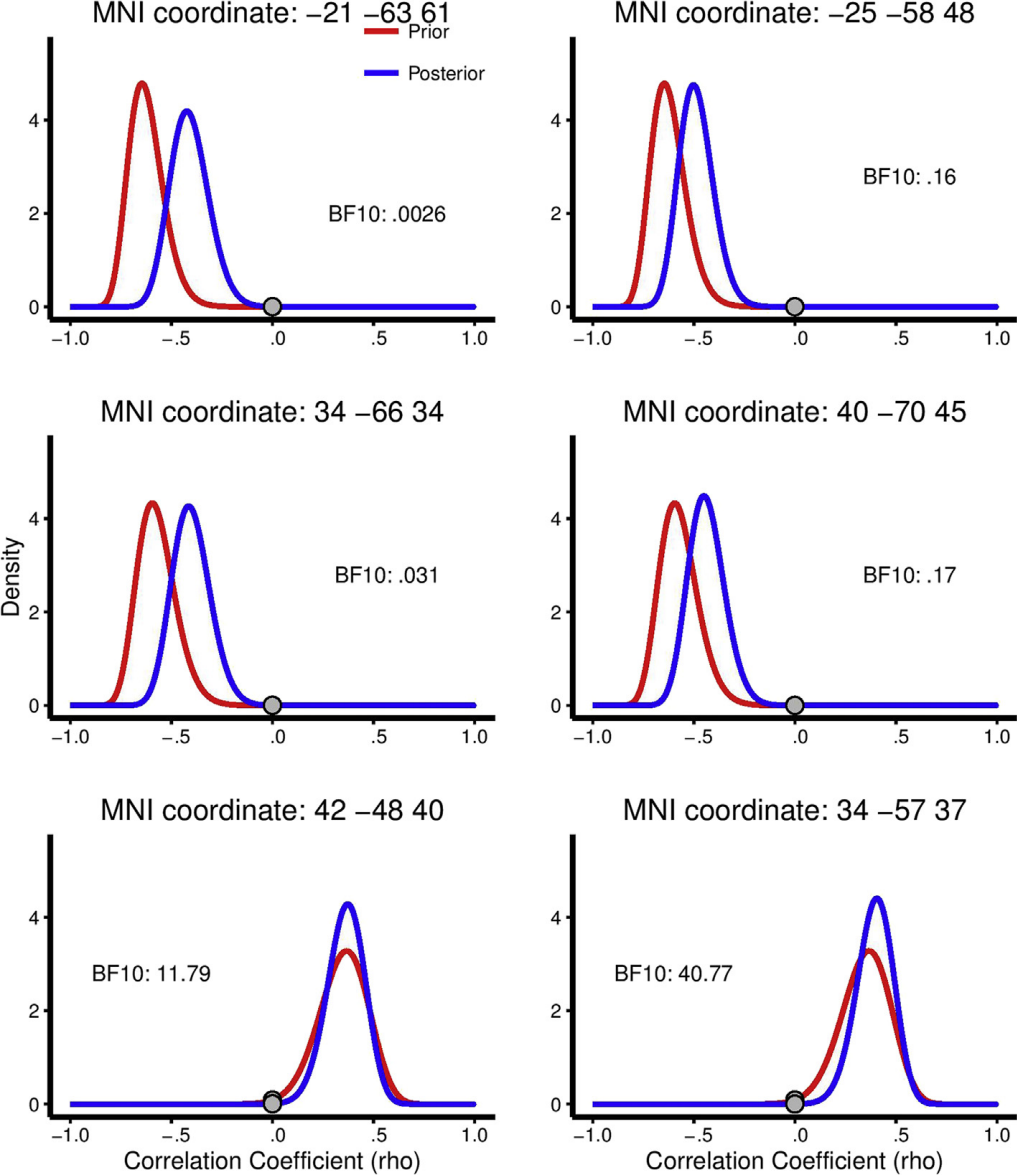
Prior and posterior distributions for GM volume correlations. Prior = red, posterior = blue. Prior distributions are based on the correlation coefficient and number of participants in the original studies. Distributions are plotted for all coordinates reported in Table 1, except for the mask ROI peak for which the original correlation was unknown. *Left*: Results for the exact coordinates reported in previous publications. *Right*: Analyses for the peaks within a spherical ROI centred on the coordinates reported in previous publications. Grey dots indicate the probability density at rho = 0 (i.e., the likelihood of no correlation) for each curve.

Second, a Bayesian hypothesis test was performed using an informative prior (based on the correlation coefficient and the number of participants in the original study). As seen in Table 1 (column 9) and Fig. 3, evidence *for* the null was moderate to extreme for the first two exact coordinates (BF = .0026 and BF = .031) and moderate for the ROI peaks (BF = .16 and BF = .17). Again, however, evidence *against* the null for the third exact coordinate was strong (BF = 11.8) and it was very strong using the ROI peak (BF = 40.8). Analysis using an informative prior was not possible for the Mask ROI condition as the correlation coefficients were unknown for voxels in the region. Overall, the analysis using informative priors lead to similar conclusions as the analysis using uniform priors.

Importantly, however, this analysis allowed us to report not only increased/decreased belief in the original finding, but also a posterior probability distribution based on both studies. The mode of this distribution is called the maximum a posteriori probability (MAP) and the 2.5 and 97.5% posterior distribution cut-off estimates are called the 95% credible interval. The MAP and the 95% credible interval are reported in Table 2 and the posterior distributions are plotted in Fig. 3.

As seen in Table 2 and Fig. 3, the MAP estimates for the first two coordinates are around |*r*| = .4 and the 95% credible interval is in the range of |*r*| = [.2; .6]. This means that although the Bayes factors reported above indicate decreased belief in the original findings for these factors, the posterior distribution indicates that if we presume that an effect is present, the most likely correlation coefficients is in the range of |*r*| = .4 ± .2 (rather than around |*r*| = .65 as the original study indicated). For the third coordinate, our MAP is very close to that of the original study, and our study has increased the belief in that estimate and made it very unlikely that the true correlation is very small.

Finally, as outlined in the Methods, posterior probability map ROI analyses were also conducted using the Savage–Dickey–Taylor approximation to the Bayes Factor. The BFs for the three spherical ROIs were 1.63, 2.25, and 5.47 respectively, the results thus being somewhat inconclusive for the ROIs based on Kanai et al. (2010) while providing moderate support for the coordinates reported by Kanai et al. (2011), which is generally consistent with the analysis above.

### 3.3 GABA MRS analyses

**Fig. 4:**
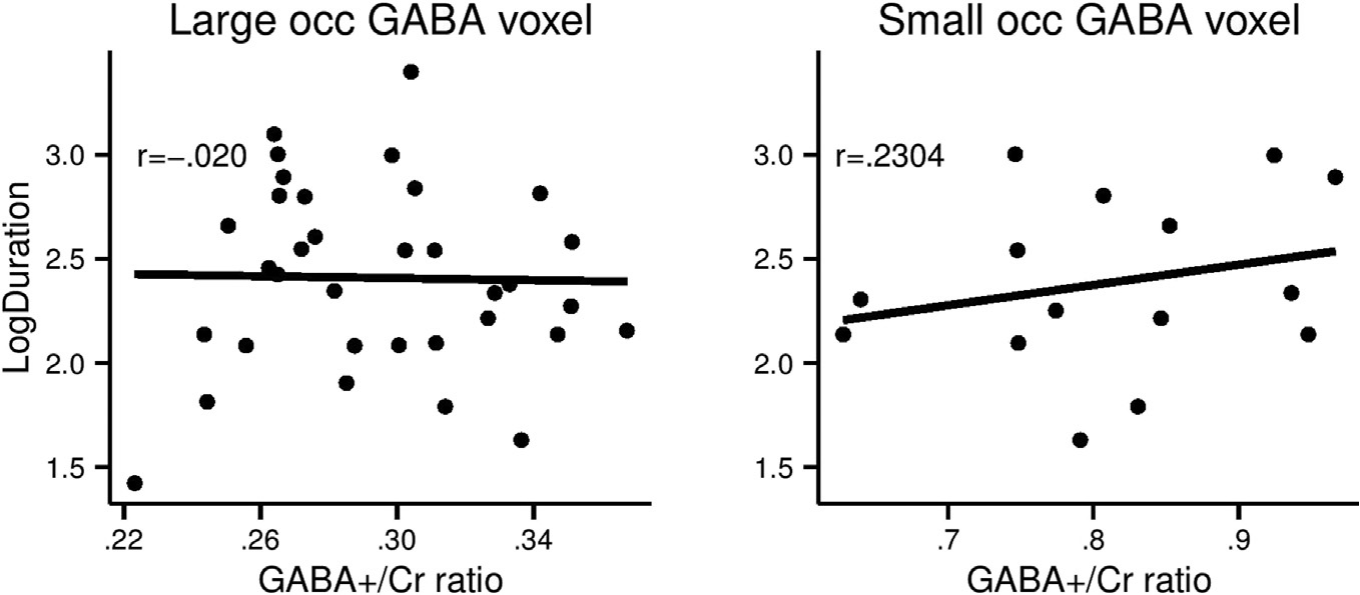
GABA correlations. Gamma aminobutyric acid (GABA) and mean log-transformed percept duration is plotted for all participants (one point representing one participant) for both MRS voxels reported in Table 2. Linear regression lines are fitted for both plots.

For the GABA MRS analysis, two voxels were used: 1) a large voxel covering a significant proportion of the occipital lobe, and 2) a small voxel covering mainly the area around the calcarine sulcus of the right hemisphere. Respectively, these voxels were around twice and half the size of the voxel used by van Loon et al. (2013). The analyses were similar to the ones performed for the VBM/percept duration relationship. Both correlations are shown in Fig. 4. For the large voxel, no relationship was found, and for the small voxel, a weak positive correlation was found between GABA/Cr ratio and SFM percept duration.

First, a one-sided Bayesian hypothesis test was performed using a uniform prior. As seen in Table 1 (column 8), moderate evidence for the null was found for the large voxel (BF = .20), whereas anecdotal evidence for the null was found for the small voxel (BF = .68). Second, a Bayesian hypothesis test was performed using an informative prior (based on the correlation coefficient and the number of participants in the original study). As seen in Table 1 (column 9) and Fig. 3, evidence for the null was strong for the large voxel (BF = .08), but still only anecdotal for the small voxel (BF = .59).

**Fig. 5:**
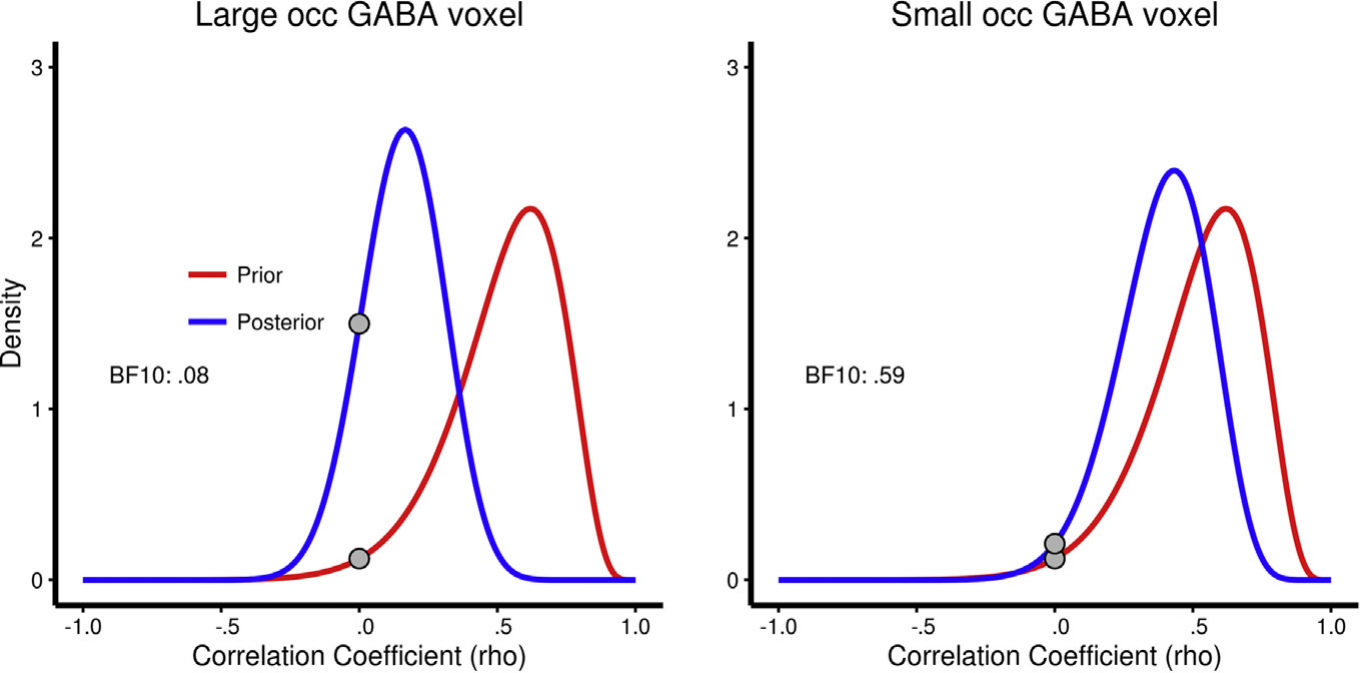
Prior and posterior distributions for MRS analysis. Prior = red, posterior = blue. Prior distributions are based on the correlation coefficient and number of participants in the original study. Distributions are plotted for both voxels reported in Table 2. Grey dots indicate the probability density at rho = 0 (i.e., the likelihood of no correlation) for each curve.

The MAP estimates along with the 95% credible intervals are reported in Table 2 and the posterior distributions are plotted in Fig. 5. As seen in Table 2 and Fig. 5, the MAP estimate for the large voxel is |*r*| = .17 and 95% of the distribution is in the range of |*r*| = [−.14; .44] whereas for the small voxel |*r*| = .44 and 95% of the distribution is in the range of |*r*| = [.03; .68]. Overall, the impact of overall occipital GABA/Cr + ratio on percept duration during SFM perception is small at best whereas the conclusion is less clear for GABA/Cr + ratio around the right calcarine sulcus, i.e., around the earliest visual areas, although the effect is likely smaller than originally reported. It may also be noted that particularly for the small occipital GABA voxel, the posterior distribution is still relatively wide (due to the low number of participants in both studies) with the 2.5% cut-off very close to 0.

### 3.4 Multiple regression analyses

Finally, we analysed the results with a set of multiple regression models in order to examine how much variability could be statistically accounted for by a combination of factors and to examine which factors contributed to such models. As GM volume values for exact and spherical ROI coordinates were highly correlated, we chose to create separate models for each set of coordinates. Similarly, as there was significant overlap between the small and large occipital GABA voxels, we created separate models for these as well. In total, four models types were created (the four combinations of exact/Sphere and small/large). The mask ROI peak coordinate was included in all four models.

**Fig. 6:**
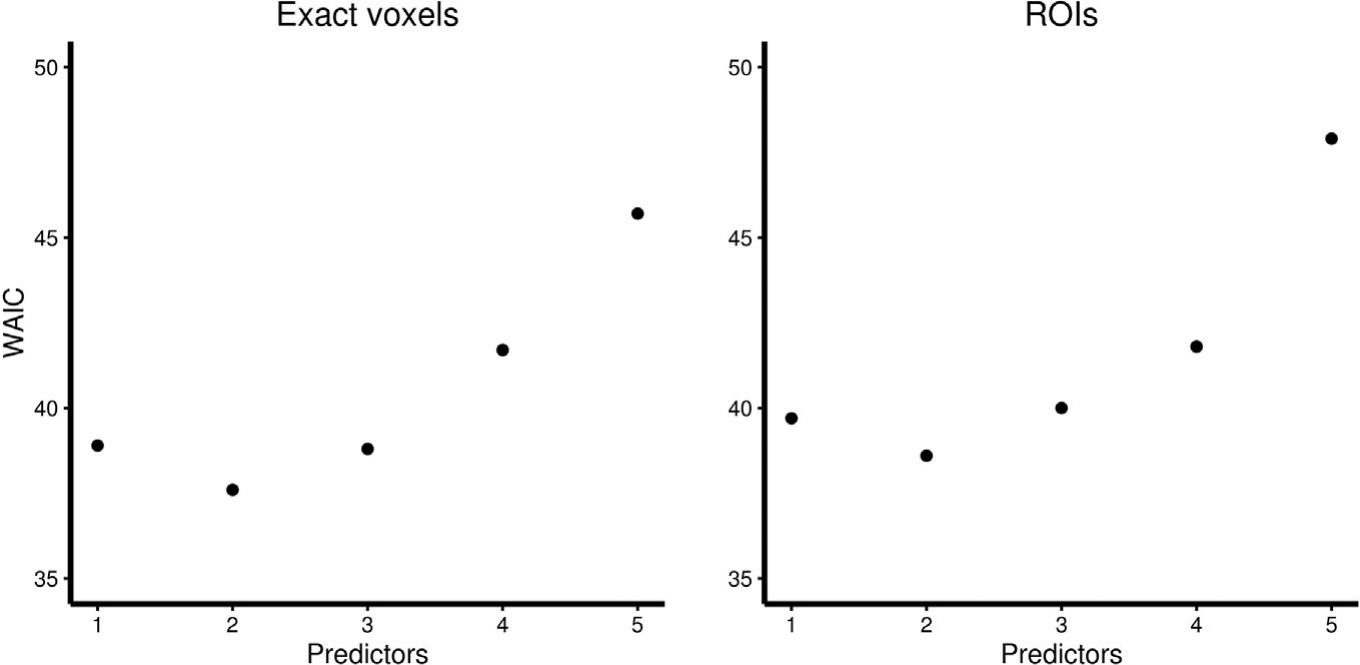
Bayesian multiple regression models. WAIC (lower is better) for multiple regression models based on the large GABA voxel data and either exact GM volume data (left) or ROI peak voxel data (right). Note that for the exact condition as well as the ROI condition, the best model had two predictors. The models are reported in Table 3.

For all four models types, model evaluation was made based on the WAIC (Watanabe, 2010). For each of these types, 32 different models can be created (combinations of the 1–5 GM volume/GABA variables), and all these models were compared. As seen in Fig. 6 and Table 3, the model with the lowest WAIC was based on 2 factors for the exact condition as well as the ROI condition. As GABA+/Cr ratio did not contribute to any model, the results are plotted for models initially including the large occipital voxel only in Fig. 6 as this model is based on the larger number of participants and thus has greater statistical power.

Both best models included the mask ROI peak, but the best exact peak model included the second GM volume peak whereas the best ROI model included the third peak. It may be noted that WAIC was low for both models in both analyses, and overall, it is thus difficult to determine, which was the better model, and we consider both relatively good. The multiple regression analysis thus lends some support to a role of the second GM volume coordinate reported in Kanai et al. (2010) for which the Pearson correlation coefficient was close to 0 in the direct replication analysis.

## 4 Discussion

We examined how well the percept duration during SFM perception was predicted from GM volume in particular areas of the SPL as well as from occipital GABA concentration (the GABA+/Cr ratio), as previous studies (Kanai et al., 2011, Kanai et al., 2010, van Loon et al., 2013) had reported such relationships.

When using frequentist statistics testing for significant correlations (reported in the Appendix), we found direct support for the role of the exact anterior SPL coordinate reported by Kanai et al. (2011), but all other tests were inconclusive (*p* > .05, corrected).

For Bayesian analyses using both uniform priors and priors informed by the correlations and number of participants in previous studies, Bayesian analyses generally provided strong support for the relationship between SFM percept duration and GM volume at the anterior coordinate reported in Kanai et al. (2011) as well as a coordinate identified by using a mask of significant results from Kanai et al. (2010) (BFs between 5 and 41). In contrast, evidence for the null was generally found for the two coordinates reported in Kanai et al. (2010) although evidence was only anecdotal in some cases (BFs between .003 and .83). Using a large voxel for GABA MRS, evidence for the null was moderate to strong depending on the prior (BF = .20 and BF = .08), but the evidence was inconclusive when using a smaller voxel placed along the calcarine sulcus of the right hemisphere (BFs = .68 and BF = .59).

Inspection of the posterior distributions for analyses using informed priors nevertheless revealed that the effect sizes should simply be adjusted downward from strong to moderate rather than to small or negligible. One exception was the relationship between percept duration and GABA+/Cr ratio in the large occipital voxel for which the correlation should be considered absent to weak. Finally, Bayesian and frequentist multiple regression models provided evidence for the potential role of GM volume in some or all examined SPL areas, but did not provide evidence in support of the role of GABA.

The fact that we cannot dismiss the originally reported effects – but instead conclude that they may simply be smaller than first thought – should not be considered a consequence of low power in the present study (or, indirectly, a low sample size). As calculated above, power was high for all tests (>.74) and even exceptionally high the majority (>.99). This may appear paradoxical, but it should be remembered that power is calculated using the original effect size, and it is this effect size we find clear evidence against in many cases. Nevertheless, ruling out a large effect size does not mean that no effect at all is present. Furthermore, the posterior distributions reflect the original as well as the new data (i.e., they are conceptually somewhat similar to meta-analyses), and it is therefore reasonable to expect that when combining similar sized samples with no effect and a large effect respectively, the combined estimate is a moderate effect.

As mentioned in the Methods section, there were some differences between the present study and the previous studies. For instance, as opposed to the Kanai et al. (2010) study, only males were included in our study as GABA levels vary across the menstrual cycle for females, and this may be a potential confound, particularly when behavioural testing and MRS is not carried out on the same day. It is an open question whether this difference could have caused the higher percept duration in the present study compared to Kanai et al. (2010), yet it seems unlikely as Kanai et al. regressed out gender as a variable in their analyses.

van Loon et al. (2013) also examined binocular rivalry (BR) and motion induced blindness (MIB) and found similar, although numerically smaller, correlations between percept duration and GABA concentration. It is highly likely that percept durations are positively correlated across the three behavioural paradigms (so that a participant with a long BR percept duration also has a long SFM percept duration). For this reason, one might assume that our results apply to these other paradigms, but since the correlation between percept duration across paradigms is unknown to us (it was not reported in the original study), we cannot know whether this is indeed the case. Thus, our data cannot be used to make inferences about the correlation between GABA and MIB/BR.

Perhaps the most important difference between the current and previous studies is that participants in the van Loon et al. (2013) study were instructed to overtly modify their percept alternation rate in half the blocks. This is particularly important as we did not find evidence in support of a role of GABA in the current study: the frequentist analysis was non-significant, the Bayes factors indicated anecdotal to strong evidence for the null (depending on the voxel), and leaving GABA out of the multiple regression models resulted in better models. It is thus an open question, whether the overt alternation trials were driving the effect in the van Loon et al. study. One reason to believe that this could be the case, is that in certain models, top-down attention is viewed as the influence of prefrontal/parietal areas upon the competition between different representations in sensory areas, resulting in the suppression of specific representations (Corbetta and Shulman, 2002, Desimone and Duncan, 1995, Desimone, 1998). Given that GABA is involved in inter-representational suppression, the role of GABA may thus be larger in cases where attention is specifically engaged in a task related manner. Consistent with this view, we have previously shown that both GM volume of the (left) SPL and occipital cortex GABA correlate with cognitive failures in daily life, and that the two factors contribute independently of one another (Sandberg, Blicher, et al., 2014). This may be caused, in part, by attentional modulation of inhibitory connections suppressing irrelevant information. A similar mechanism might be involved in bistable perception where the success of attempt to reverse perception is dependent on parietal GM characteristics as well as occipital GABA (inhibitory) characteristics.

The specific correlation coefficients obtained in studies such as the present, depend on the method for selecting target areas. For our GABA analysis, the large voxel was selected as it covered large parts of the occipital lobe, but could still be centred on the calcarine sulcus while not extending into other lobes, the cerebellum or the skull. Pilot tests also showed that data quality was very high for this voxel (estimated by visual inspection and later confirmed by SNR calculation). The small voxel was selected to obtain data from an area closely following the calcarine sulcus of the right hemisphere, including little or no cerebrospinal fluid between hemispheres, thus making it likely to obtain a better measure of relative V1 GABA concentration. Different voxel positions/sizes could in principle have caused slightly different results, but it is important to note that moderate changes in voxel position have only a minor impact on GABA estimations (Near et al., 2014).

For GM volume analysis, multiple methods are possible for selecting target areas. As mentioned above, simply selecting the peak coordinate of a previous study is likely to result in underestimation of the peak correlation due to small spatial differences across samples. This problem is also present if instead a mean correlation is selected across all voxels showing a significant correlation in a previous study (Kanai, 2016). On the other hand, using a multivoxel (e.g., spherical) ROI centred on the coordinates of the peak correlation in a previous study is likely to overestimate the true peak correlation. This is because random variability will cause voxels to show correlations normally distributed around the true mean. For example, even if the true correlation is 0 in a sample of 37 participants, 10% of all tested voxel will have |*r*| > .27 by chance alone. This is not a problem in analyses correcting for multiple comparisons if only the result of the significance test is used, but the reported peak correlation coefficient is likely to be inflated. In the present study, we thus used a method that we expected to be too conservative and another we expected to be too liberal. The true correlations are thus expected to be somewhere between the estimates of the two methods. Importantly, as seen in Table 2, the MAP estimates of the two methods were relatively similar, indicating that the methods did not provide estimates so different that comparison is meaningless.

Taken together, the current study contributes new data on the relationship between perceptual alternations for ambiguous stimuli and GM volume as well as occipital GABA. Whereas we find no direct support for the role of occipital GABA, the combined data of the two studies is nevertheless insufficient to support direct rejection of potential relationship. However, if a relationship is indeed present, it appears less strong than originally reported. For posterior SPL GM volume, the conclusion is somewhat similar although multiple regression results do provide some evidence to support the original finding. In contrast, all performed analyses provide evidence for a role of the anterior, right hemisphere SPL, and the Bayesian analyses provide support that the effect size is as originally reported.

Evidence for a role of the anterior parietal lobe in reversals during bistable perception was reported almost 20 years ago (Lumer et al., 1998), and the reported coordinates are strikingly similar to the peak coordinates in our data ([30, −54, 54] *vs* [34, −57, 37]. However, this and more recent studies have also found evidence for the involvement of the occipital lobe (e.g., Britz & Pitts, 2011). As mentioned in the Introduction, we recently demonstrated that gradual changes in stimulus-specific MEG activity in the occipital lobe was detected long before perceptual reversals, but changes in parietal activity were only detected immediately before a reversal (Sandberg et al., 2014a, Sandberg et al., 2014b). This indicates that the parietal lobe may be involved in the initiation and execution of perceptual reversals, but that the decision when to do this could be partially determined by sensory cortex characteristics. Unfortunately, the sample size of the present study makes it suitable only for tests of very specific hypotheses as the statistical power is low for analyses at the whole brain or lobe level. Nevertheless, we propose that the hypothesis is tested in experiments including a much larger number of participants.

One very specific hypothesis for the relative role of the sensory cortex and the parietal lobe in perceptual reversals is that the sensory cortex performs the initial sensory processing while the parietal lobe seeks to infer the most likely environmental cause that gave rise to the input by generating forward models or hypothesis (Kanai et al., 2011). This has been worded in a hierarchical Bayesian network theory framework, and it has been proposed that while the posterior parietal lobe generates the prediction signal, the anterior parietal lobe generates the prediction error signal. This would explain the proposed opposite role of the two areas in that weak predictions may lead to an increased reversal frequency (shorter percept durations) while weak error signals could lead to a decreased reversal frequency (longer percept durations) (Kanai et al., 2011). This hypothesis has received some support from a dynamic causal modelling (DCM) fMRI study demonstrating that the bottom-up coupling between V5/MT, pSPL and aSPL predicted mean percept duration during SFM (Megumi, Bahrami, Kanai, & Rees, 2015), and it is consistent with the parameter values reported in our ROI multiple regression model.

## Figures and Tables

**Table A1:** Main results: Frequentist analysis. Original MNI coordinates for GM volume findings from Kanai et al., 2010, Kanai et al., 2011 are reported in column 1, rows 1–6. Rows 7–8 reports replication tests based on the van Loon et al. (2013) study. Column 2 reports the condition: The ROI condition uses spherical ROI with a radius of 15 mm centred on the original peak. The exact condition uses the exact original peak coordinate. The Mask condition uses a mask consisting of all significant voxels in Kanai et al. (2010). The large and small conditions use large and small voxels for GABA MRS, both centred on the calcarine sulcus. *p* values are based on one-sided hypothesis tests. For spherical and Mask ROI analyses, *p* values are corrected using the family-wise error rate.

Original	Condition	Replication coordinate	N_original_	N_replication_	*r*_original_	*r*_replication_	*p*_corrected_
−21 −63 61	Exact	−21 −63 61	52	37	−.65	.0130	.530
−21 −63 61	Sphere	−25 −58 48	52	37	−.65	−.220	.585
34 −66 34	Exact	34 −66 34	52	37	−.60	−.0875	.303
34 −66 34	Sphere	40 −70 45	52	37	−.60	−.196	.520
42 −48 40	Exact	42 −48 40	52	37	.37	.3856	.0092
42 −48 40	Sphere	34 −57 37	52	37	.37	.4644	.073
Multiple	Mask	21 −58 66	52	37	N/A	.4845	.091
Occ GABA	Large		14	34	.636	−.0203	.54
Occ GABA	Small		14	15	.636	.2304	.20

**Fig. A1:**
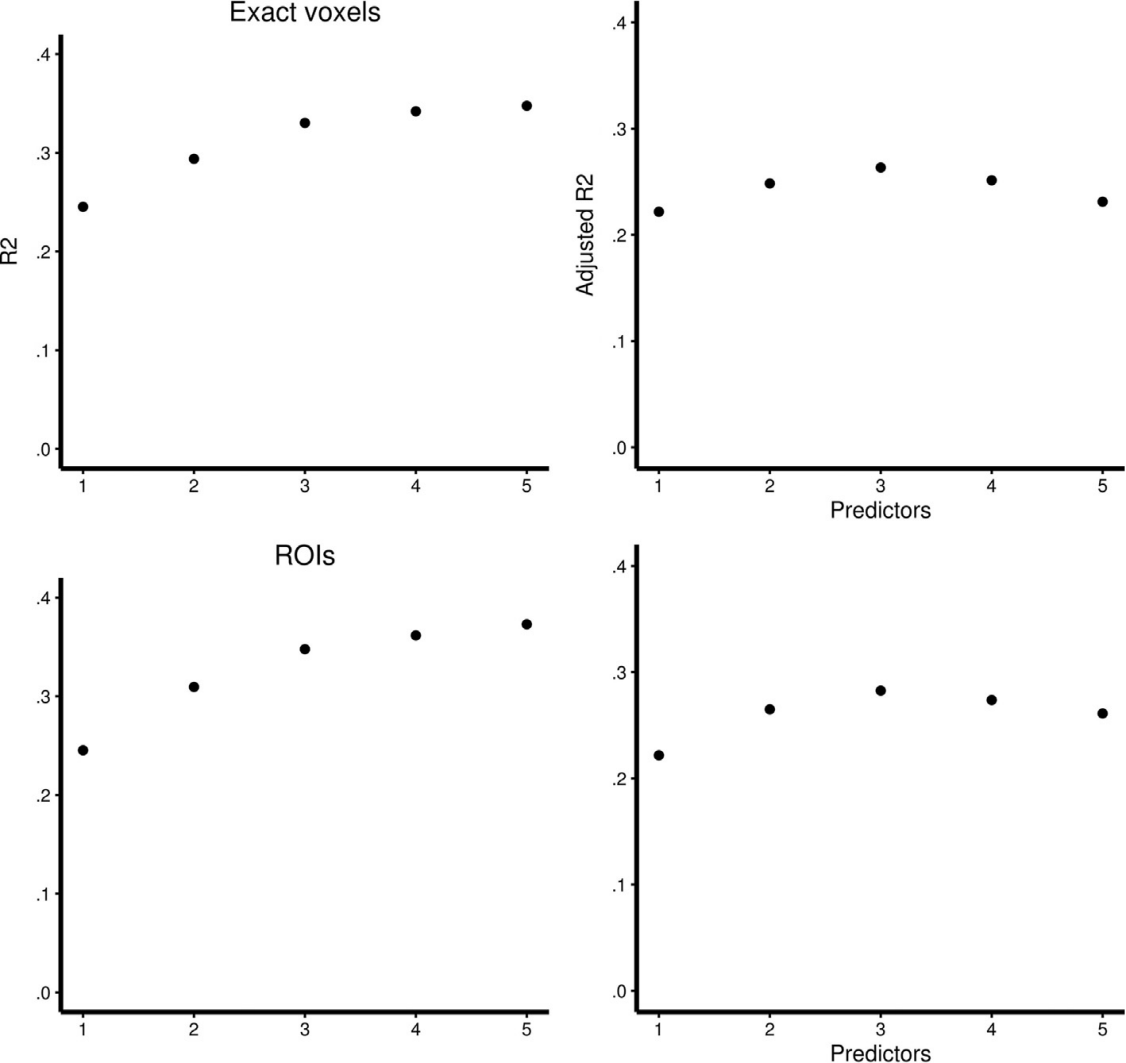
Frequentist multiple regression models. *r*^2^ (top) and adjusted *r*^2^ (bottom) for multiple regression models based on the large GABA voxel data and either exact GM volume data (left) or ROI peak voxel data (right). Note that for the exact condition as well as the ROI condition, the best model (with the highest adjusted *r*^2^) had three predictors. The models are reported in Table A2.

**Table A2:** Frequentist multiple regression parameters. The parameter values for the models with the highest adjusted *r*^*2*^. The model using the exact coordinates reported in previous studies is presented at the top; the model using peak correlations within an ROI is presented at the bottom. The peak coordinate from the mask ROI condition [21 −58 66] is included in both models. Inclusion of GABA+/Cr ratio resulted in a lower *r*^*2*^ for both model types and is thus not presented in the table.

Model	Parameter	Value
Exact	Intercept	2.40
	−21 −63 61	−1.73
	34 −66 34	0
	42 −48 40	1.27
	21 −58 66	3.41
ROI	Intercept	2.38
	−25 −58 48	0
	40 −70 45	−1.30
	34 −57 37	2.04
	21 −58 66	2.67

**Table 1:** Main results. Original MNI coordinates for GM volume findings from Kanai et al., 2010, Kanai et al., 2011 are reported in column 1, rows 1–6. Rows 7–8 reports replication tests based on the van Loon et al. (2013) study. Column 2 reports the condition: The ROI condition uses spherical ROI with a radius of 15 mm centred on the original peak. The exact condition uses the exact original peak coordinate. The Mask condition uses a mask consisting of all significant voxels in Kanai et al. (2010). The large and small conditions use large and small voxels for GABA MRS, both centred on the calcarine sulcus. Bayes factors (BF) are reported in favour of the original hypothesis using uniform one-sided priors and using informative priors based on the number of participants (N) and correlation coefficient (*r*) reported in the original studies.

Original	Condition	Replication coordinate	N_original_	N_replication_	*r*_original_	*r*_replication_	BF_10_uniform_	BF_10informative_
−21 −63 61	Exact	−21 −63 61	52	37	−.65	.0130	.20	.0026
−21 −63 61	Sphere	−25 −58 48	52	37	−.65	−.220	.83	.16
34 −66 34	Exact	34 −66 34	52	37	−.60	−.0875	.32	.031
34 −66 34	Sphere	40 −70 45	52	37	−.60	−.196	.68	.17
42 −48 40	Exact	42 −48 40	52	37	.37	.3856	5.48	11.8
42 −48 40	Sphere	34 −57 37	52	37	.37	.4644	20.6	40.8
Multiple	Mask	21 −58 66	52	37	N/A	.4845	15.4	N/A
Occ GABA	Large		14	34	.636	−.0203	.20	.08
Occ GABA	Small		14	15	.636	.2304	.68	.59

**Table 2:** Posterior distribution characteristics. Maximum posteriori probability (MAP) estimate as well as the 2.5 and 97.5% cut-off values (95% credible interval) for the posterior distribution of *r* is plotted for all GM volume and GABA measures for which analyses could be performed using an informative prior. Note that although evidence was found in support of the null hypothesis for several factors, posterior distributions indicate that effect sizes may simply be smaller than originally reported and that the original findings thus may not be false positives.

Original	Condition	MAP estimate	2.5% cut-off	97.5% cut-off
−21 −63 61	Exact	−.424	−.584	−.211
−21 −63 61	ROI	−.496	−.637	−.303
34 −66 34	Exact	−.414	−.573	−.207
34 −66 34	ROI	−.450	−.600	−.251
42 −48 40	Exact	.373	.169	.533
42 −48 40	ROI	.406	.205	.56
Occ GABA	Large	.167	−.144	.437
Occ GABA	Small	.432	.028	.679

**Table 3:** Bayesian multiple regression parameters. The parameter values for the models with the lowest (better) WAIC. The model using the exact coordinates reported in previous studies is presented at the top; the model using peak correlations within an ROI is presented at the bottom. The peak coordinate from the mask ROI condition [21 −58 66] is included in both models. Inclusion of GABA+/Cr ratio resulted in a higher (worse) WAIC for both model types and is thus not presented in the table.

Model	Parameter	Value
Exact	Intercept	0
	−21 −63 61	0
	34 −66 34	−1.30
	42 −48 40	0
	21 −58 66	3.91
ROI	Intercept	0
	−25 −58 48	0
	40 −70 45	0
	34 −57 37	2.27
	21 −58 66	2.52

## 5 Appendix

This appendix reports frequentist analyses.

### 5.1 VBM analyses

As for the Bayesian analyses, replication analyses were performed using the exact peak coordinate reported in previous publications coordinates (no correction) as well as spherical and mask ROIs (family-wise error corrected). The spherical ROI condition was advocated by Kanai (2016) and used in previous publications (Kanai et al., 2011, Sandberg et al., 2014b): Hypothesis tests corrected for multiple comparisons for the spherical and mask ROIs analyses. For the exact coordinates and the spherical ROIs, the tests were one-tailed (as the direction was known) whereas the test was two-tailed for the mask ROI. The results are presented in Table 1.

The null hypothesis could only be rejected for the anterior GM volume peak at the exact coordinate (*p* = .0092) (Table 2, column 5), but not at the ROI peak (*p* = .073). All other tests were non-significant, i.e., inconclusive. The results are relatively consistent with the Bayesian analyses providing evidence against the null for the third (anterior) peak whereas the results are inconclusive for the remaining conditions.

### 5.2 GABA analyses

Replication analyses (one-tailed, uncorrected) are reported in Table A1. Both tests were non-significant, i.e., inconclusive.

### 5.3 Multiple regression analyses

Multiple regression analyses were also conducted. As in the Bayesian analyses, four models were created (the four combinations of exact/Sphere and small/large). The mask ROI peak coordinate was included in all four models.

For all four models, model selection was made based on adjusted *r*^2^. As seen in Fig. A1 and Table A2, the model with the highest adjusted *r*^2^ was based on 3 factors for the exact condition as well as the ROI condition, but the first model included the first GM volume peak whereas the latter included the second peak. Both models included the third GM volume peak and the mask ROI peak. As GABA+/Cr ratio did not contribute to any model, the results are plotted for models initially including the large occipital voxel only in Fig. A1 as this model is based on the larger number of participants and thus has greater statistical power. As mentioned earlier, the model based on ROI values is likely to overestimate the true correlation whereas the model based on exact coordinates is likely to underestimate the true correlation, but the estimates of inter-individual variance explained (*r*^2^) were nevertheless highly similar. The best estimate for the percent of variance explained is thus likely to be somewhere between the estimates for the two models: 33–35%.

In summary, this analysis shows that a significant amount of variability in behaviour (i.e., percept duration during SFM perception) can be explained by a combination of the factors that were examined. Whereas inclusion of GABA+/Cr ratio resulted in a worse model when taking into account the increased complexity, inclusion of GM volume at all coordinates that were examined resulted in better models for either the exact peak or ROI conditions. This finding is important as it provides evidence for 1–2 GM volume coordinates at which the Pearson correlation coefficient was close to 0. This analysis is relatively consistent with the Bayesian analysis, which also provided evidence for at least one coordinate for which the Pearson correlation coefficient was close to 0.
